# Synergy between hemagglutinin 2 (HA2) subunit of influenza fusogenic membrane glycoprotein and oncolytic Newcastle disease virus suppressed tumor growth and further enhanced by Immune checkpoint PD-1 blockade

**DOI:** 10.1186/s12935-020-01476-5

**Published:** 2020-08-07

**Authors:** Seyed Mohammad Miri, Mir Saeed Ebrahimzadeh, Elahe Abdolalipour, Mahsa Yazdi, Hassan Hosseini Ravandi, Amir Ghaemi

**Affiliations:** 1 Sharif University of Technology, Tehran, Iran; 2 Department of Microbiology, Golestan University of Medical Sciences, Gorgan, Iran; 3 Department of Virology, Pasteur Institute of Iran, P.O.Box: 1316943551, Tehran, Iran; 4 Shefa Neuroscience Research Center, Khatam Alanbia Hospital, Tehran, Iran

**Keywords:** Cervical cancer, Fusogenic membrane glycoprotein, Newcastle disease virus, PD-1 blockade, Influenza hemagglutinin 2

## Abstract

**Background:**

Newcastle disease virus (NDV) has shown noticeable oncolytic properties, especially against cervical cancer. However, in order to improve the spread rate and oncotoxicity of the virus, employment of other therapeutic reagents would be helpful. It has been shown that some viral fusogenic membrane glycoproteins (FMGs) could facilitate viral propagation and increase the infection rate of tumor cells by oncolytic viruses. Additionally, immune checkpoint blockade has widely been investigated for its anti-tumor effects against several types of cancers. Here, we investigated for the first time whether the incorporation of influenza hemagglutinin-2 (HA2) FMG could improve the oncolytic characteristics of NDV against cervical cancer. Next, we added anti-PD-1 mAb to our therapeutic recipe to assess the complementary role of immune checkpoint blockade in curbing tumor progression.

**Methods:**

For this purpose, TC-1 tumor cells were injected into the mice models and treatment with NDV, iNDV, HA2, NDV-HA2, iNDV-HA2 began 10 days after tumor challenge and was repeated at day 17. In addition, PD-1 blockade was conducted by injection of anti-PD-1 mAb at days 9 and 16. Two weeks after the last treatment, sample mice were sacrificed and treatment efficacy was evaluated through immunological and immunohistochemical analysis. Moreover, tumors condition was monitored weekly for 6 weeks intervals and the tumor volume was measured and compared within different groups.

**Results:**

The results of co-treatment with NDV and HA2 gene revealed that these agents act synergistically to induce antitumor immune responses against HPV-associated carcinoma by enhancement of E7-specific lymphocyte proliferation, inducement of CD8^+^ T cell cytotoxicity responses, increase in splenic cytokines and granzyme B, decrease in immunosuppressive cytokines and E6 oncogene expression, and upregulation of apoptotic proteins expression, in comparison with control groups. Moreover, incorporation of PD-1 blockade as the third side of our suggested therapy led to noticeable regression in tumor size and augmentation of cytokine responses.

**Conclusions:**

The invaluable results of synergy between NDV virotherapy and HA2 gene therapy suggest that tumor-selective cell killing by oncolytic NDV can be enhanced by combining with FMG gene therapy. Moreover, the adjunction of the PD-1 blockade proves that checkpoint blockade can be considered as an effective complementary therapy for the treatment of cervical cancer.

## Background

Cervical cancer as the 4th prevalent gynecological cancer type and one of the main cancer-related reasons for mortality among women is in demand for worldwide attention of all experts from gynecologists to cancer therapy developers [1]. Conventional therapeutic approaches such as surgery, chemotherapy, and radiotherapy have been widely used to overthrow this widespread malignancy. However, statistics about the incidence of many thousands of newfangled cases diagnosed with cervical cancer and the huge number of deaths among previously diagnosed patients in 2018 clarify the inefficiency of present cervical cancer prevention and treatment methods [2]. To address the shortcomings of present therapeutic methods and along with advancements in synthetic biology, immunotherapy and oncolytic virotherapy have emerged as alternative or complementary cervical cancer treatments [3]. Regarding immunotherapy techniques, the employment of HPV vaccines, adoptive T-cell therapy, and immune checkpoint blockade have investigated [4, 5]. For example, immune checkpoint blockade of programmed death-1 (PD-1), known as the immunoinhibitory receptor that contributes to immune evasion of various solid tumor cells, has been investigated as an adjuvant therapy against cervical cancer models [6]. Moreover, pembrolizumab, the highly selective humanized anti-PD-1 monoclonal antibody, is under investigation in phase II clinical trial for the treatment of advanced cervical cancer [7]. However, the effectiveness of monotherapy with immune checkpoint blockade in clinical trials is doubtful and in some cases, the results are not satisfactory enough [8]. Besides these dissatisfactory approaches, oncolytic virotherapy with distinctive characteristics has shown promising results against cervical cancer [3]. Direct oncolysis along with the provocation of host systemic innate and adoptive immune responses introduce oncolytic virotherapy as one of the treatment methods with the most optimistic results in the treatment of human cancers [9].

Newcastle disease virus is an avian paramyxovirus, which has been shown to induce oncolytic activity against human tumors [10]. Despite its wild-type, cell culture-adapted strains of NDV such as LaSota can be used as a safe oncolytic virus with minimum virulence activity [11]. The major concern with the employment of oncolytic viruses is a limitation related to their spread through the tumor microenvironment [12]. To address this deficiency, scientists have focused on the natural evolutionary ability of some viral genes in the creation of syncytia between infected cells and nearby uninfected ones [13]. It has been shown that FMG facilitates the spread of the virus and increases the potential of virotherapy to destruct more malignant cells, even at lower doses of the virus [12]. In addition, FMGs are able to act as a synergistic approach to enhance the anti-tumor effects of other therapies [12, 14–17]. In the present study, we investigated whether coupling the oncolytic NDV virotherapy with influenza hemagglutinin-2 as FMG enhanced the anti-tumor efficacy of cervical cancer therapy. Moreover, it was evaluated whether there is a synergy between PD-1 blockade and HA2-assisted NDV oncolytic virotherapy.

## Methods

### Virus and cell lines

The LaSota NDV strain used in this study was prepared from the Razi Institute of Serum and Vaccine Research Center (Alborz, Iran). The strain was propagated in the allantoic cavity of 9- to 11-day-old SPF embryonated chicken eggs, and all allantoic fluid samples were harvested and stored at − 80 °C until use. The titer of the virus was determined using Embryo Infectious Dose 50 (EID50) assay. In order to inactivate NDV, the sample was exposed to the UV radiation [18] and the result was confirmed in Vero cells by plaque-forming assay [19]. We did not observe any plaque in Vero cells formed by UV-inactivated NDV. EID50 is commonly used as a titration unit of the NDV. For EID50 to PFU conversion, we used EID/50 ∼ 0.7PFU conversion factor [20].

The murine TC-1 cell line, expressing E6 and E7 oncoproteins from HPV-16, was purchased from the National Cell Bank (Pasteur Institute of Iran). Briefly, TC-1 cells were cultured in complete RPMI 1640 media (Gibco BRL, Gaithersburg, MD, USA) containing 10% fetal bovine serum (FBS) (Gibco, Rockville, MD), 100 U/mL of penicillin, 100 μg/mL of streptomycin, and 0.4 mg/mL G418 (all from GIBCO, UK), 0.5 mM sodium pyruvate (Sigma Aldrich, Germany), and 2 mM  l-glutamine. The EL4 cell line (murine T-cell lymphoma of haplotype H-2b derived from C57BL/6 mice) was cultured in RPMI 1640 supplemented with 10% FBS.

### Construction of the HA2 Vector

**Fig. 1:**
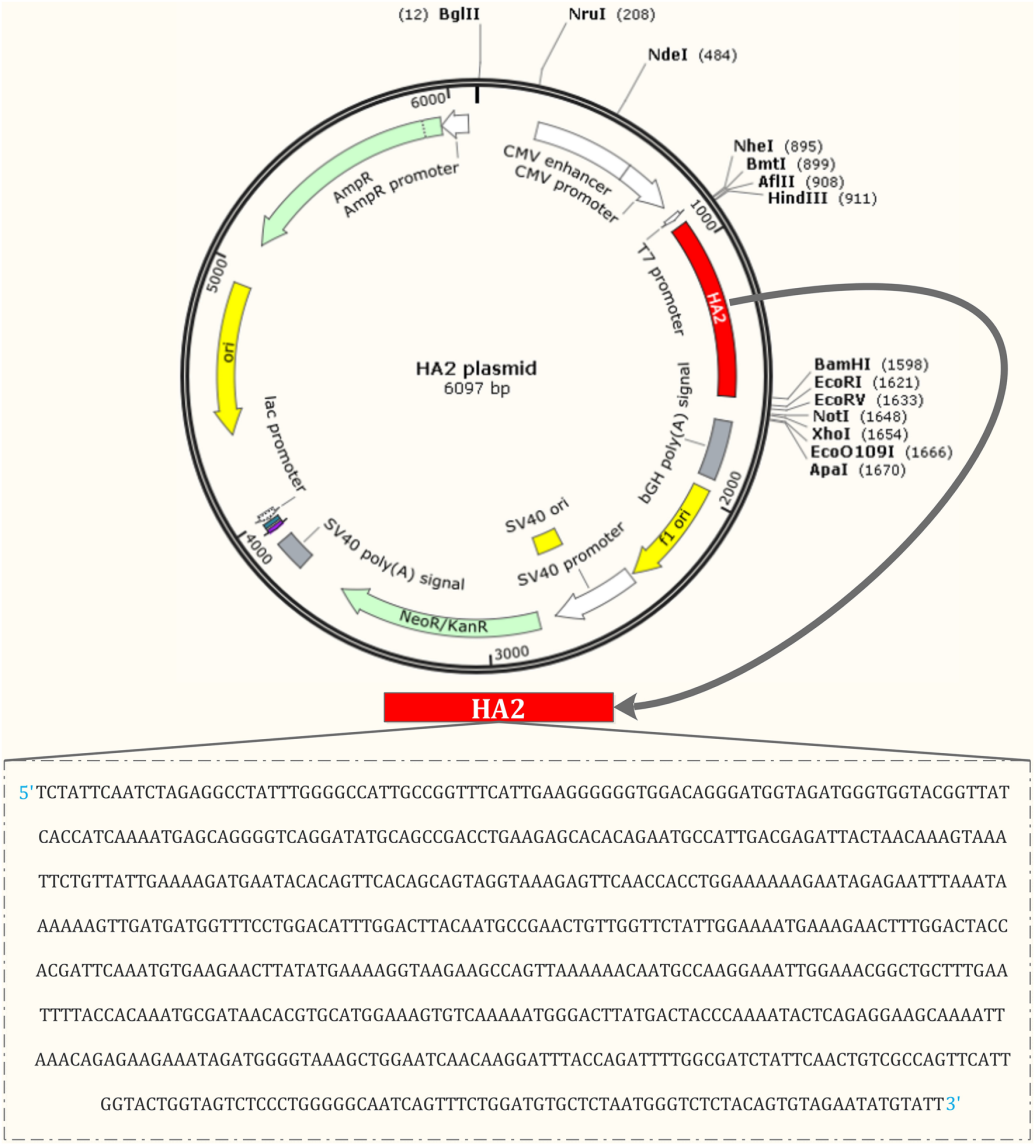
Schematic map and details of pcDNA3-HA2 expression plasmid

The generation of pcDNA3-HA2 expression plasmid has been described previously [21] (Fig. 1). Plasmid constructs were confirmed by DNA sequencing and expression. Large-scale preparations of endotoxin-free plasmids and vector control plasmid (pcDNA3.1) were obtained using the EndoFree® Plasmid Maxi Kit (Qiagen, Hilden, Germany).

### Animal studies

**Fig. 2:**
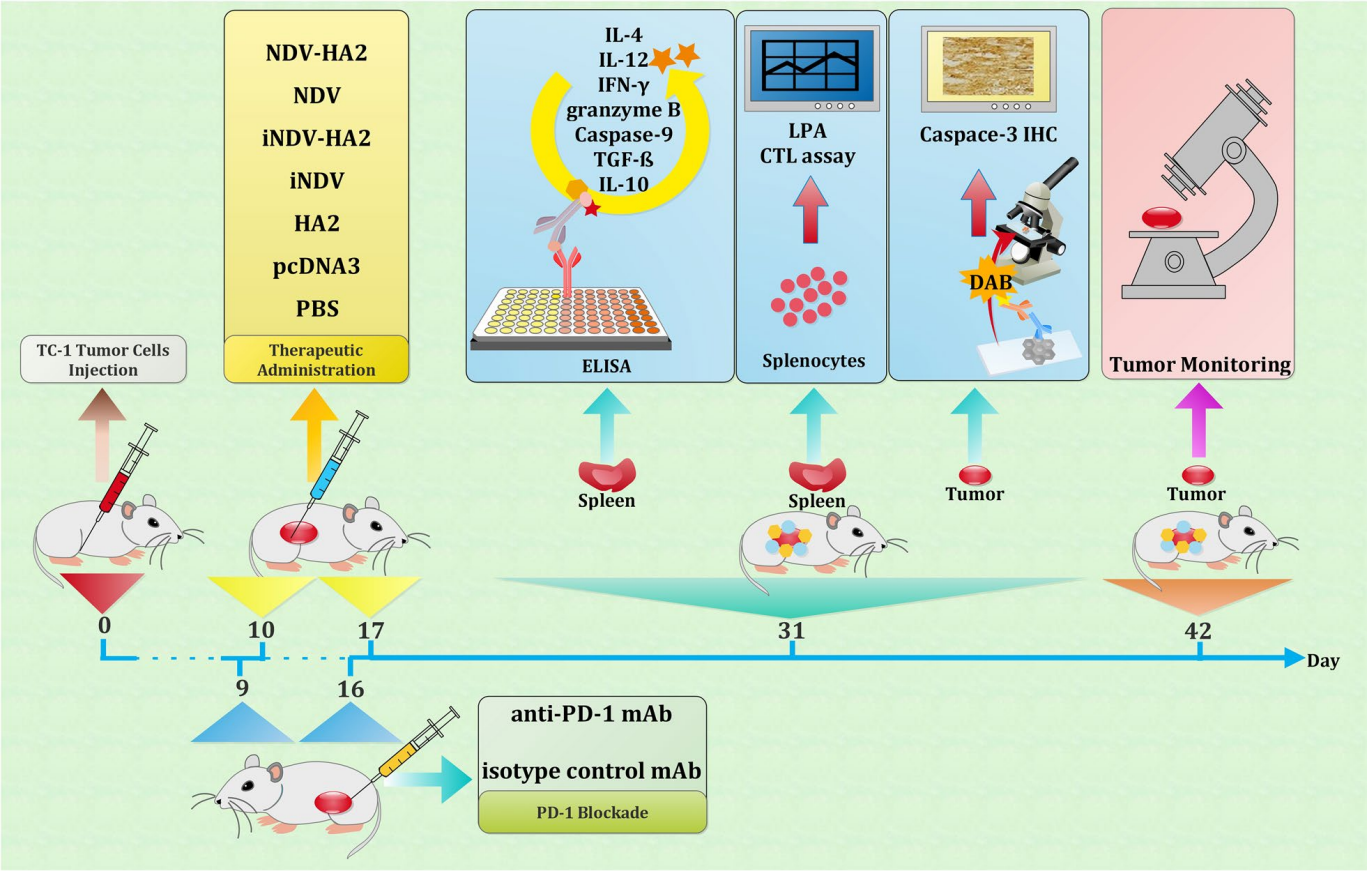
Schematic overview of all experimental procedures. The blue arrow indicates the time schedule of experiments from day 0 (subcutaneous injection of TC-1 tumor cells) to day 42 (final monitoring and evaluation of tumor)

All experiments were performed according to the Ethical Committee for the use and care of laboratory animals of Pasteur Institute of Iran (ethics number: IR.IUMS.FMD.REC 1396.9321540001). Six- to eight-week-old female C57BL/6 (H2b) mice were obtained from the Pasture Institute of Iran. The mice were adapted to the environment for 1 week before the experiment with sufficient supply of food and water and were kept in a 12–12 light period. Animals treated with the active and inactivated NDV alone or in combination with HA2 plasmid. ***In vivo*** tumor induction was conducted through subcutaneous injection of 7 × 10^5^ TC-1 tumor cells per mouse into the right flank area of the mice on day 0 and then they randomly divided into seven different groups (10 mice/group). Ten days after tumor injection, mice were treated peritumorally with NDV (2 × 10^7^ PFU/100 µl activated NDV in 100 µl of PBS), iNDV (2 × 10^7^ PFU/100 µl inactivated NDV), 100 µg influenza HA2 plasmid alone or NDV-HA2 (2 × 10^7^ PFU/100 µl activated NDV + 100 µg influenza HA2 plasmid), iNDV-HA2 (2 × 10^7^ PFU/100 µl inactivated NDV + 100 µg influenza HA2 plasmid), PBS (100 µl), and pcDNA3 (100 µg), twice at one-week intervals. Tumor growth and survival were monitored two to three times a week. Afterward, mice were monitored twice a week by inspection and palpation. Tumor size was evaluated by measuring the length (i.e., the longest dimension) and width (i.e., the shortest dimension) by means of electronic calipers. Tumor volume was calculated by the simplified formula of a rotational ellipse (*l* × *w*^2^ × 0.5). Three mice per group were sacrificed two weeks after the second treatment (day 31), the spleens were removed aseptically and prepared to determine the immune responses of splenic lymphocytes. The tumor tissues also were removed, weighed, tabulated from the mice, fixed in buffered formalin, and processed for immunohistochemical (IHC) analysis of the caspase-3 expression. The schematic depiction of all experimental procedures is presented in Fig. 2.

### PD-1 blockade

In a parallel study and one day prior to all of the treatments, Tc-1-transplanted mice (n = 03) were injected intra-peritoneally with 100 mg of anti-mouse PD-1 after reconstitution in PBS. The control groups received an isotype-matched IgG control reconstituted and injected in a similar fashion. Rat anti-mouse PD-1 mAb (clone RMP1-14) and rat IgG2a isotype control were purchased from BioXcell. Two weeks following the final blockade and treatment (31 days post tumor challenge), E7-specific IFN- γ and IL-12 responses (n = 03) were determined (as described in cytokine secretion assay). The tumor volume was also monitored up to 6 weeks after tumor challenge (n = 07) as described in the previous section.

### Lymphocyte proliferation assay (LPA)

In order to investigate whether combinatorial NDV virotherapy and HA2 gene therapy could induce antigen-specific cell-mediated immunity, lymphocyte proliferation assay was performed in vitro. In this assay, the capability of re-stimulated splenocytes in converting tetrazolium to insoluble purple formazan was evaluated. Two weeks after last treatment, cells were seeded at 2 × 10^5^ cells/well in 96-well round-bottom plates containing RPMI-1640 supplemented with 10% FBS, 1% l-glutamine, 1% HEPES, and 0.1% penicillin/streptomycin (in triplicate), followed by 72 h incubation at 37 °C in a 5% CO_2_ incubator in the presence of 1 µg/ml E7-specific H-2Db CTL epitope (1 µg/ml, Biomatik, Ontario, Canada, > 99% purity), PHA (for positive control), and medium alone (for negative control). Afterward, the supernatants were removed and the pellets were solubilized in 100 µl dimethyl sulfoxide, attempting to eliminate the possibly produced crystals of formazan. Plates were read at a wavelength of 540 nm and the stimulation index was used for interpretation of the results. This index was obtained as follows in which Cs stands for OD values of stimulated cells, Cu stands for relative cell numbers of unstimulated cells, and SI stands for stimulation index:

$$ {\text{SI }} = {\text{ }}({\text{Cs}} - {\text{Cu}})/{\text{Cu}}. $$

### In vitro cytotoxic activity

In order to evaluate the effect of the HA2 gene on oncolytic NDV-based induction of cytotoxic immune responses by activating antigen-specific cytotoxic T lymphocytes, in vitro cytotoxic T lymphocyte (CTL) assay, by measuring the lactate dehydrogenase (LDH) release, was performed. Two weeks after the last treatment, a single-cell suspension of splenocytes (applicable as effector cells) was prepared. For the preparation of the target cells, EL4 cells were pulsed with 1 µg/ml E7-specific H-2Db CTL epitope. An exact viable number of 4 × 10^4^ EL4 cells in a volume of 100 µl (as target cells) were co-cultured with effector cells (100 µl) at 50:1 effector-to-target cell (E/T) ratios, in which a maximal release of LDH was observed. After centrifugation, the supernatants (50 µl/well) were transferred to 96-well plates and CTL activity was measured [22].

### Cytokine ELISA assay

Two weeks after the second treatment, the spleen of the mice (n = 03) were isolated and mononuclear cells from spleen of immunized mice were seeded at a concentration of 2 × 10^5^ cells/well in 24-well plates (TPP, Switzerland) and cultured for three days in RPMI1640 supplemented with 10% FBS, 1%  l-glutamine, 1% HEPES, 2.5 mM 2-mercaptoethanol, and stimulated with E7-specific H-2Db CTL epitope at a concentration of 1 µg/ml (Biomatik, Ontario, Canada, > 99% purity) at 37 °C and 5% CO_2_. The cell supernatants were collected after 48 h and the secretion of IL-4, IFN-γ, IL-12, and granzyme B in the supernatant were evaluated by commercially available ELISA kits (R&D Systems Inc., Minneapolis, Minn, USA) following the manufacturer’s instructions. All tests were performed in triplicate and the plates were read at optical density (OD) 450 nm.

### Intratumoral caspase-9 activity and IL-10 and TGF-ß secretion assay

Intrinsic apoptosis is one of the pathways that may be induced by oncolytic NDV. In order to evaluate the impact of HA2 on NDV-induced intrinsic apoptosis, caspase-9 activity in the tumor microenvironment was measured by caspase ELISA kit (Abcam, Cambridge, MA, USA) two weeks after the second treatment. Also, at the same time, the levels of immunosuppressive cytokines of interleukin-10 (IL-10) and transforming growth factor-β (TGF-β) in the tumor microenvironment were examined. Briefly, to measure the caspase-9 activity, the tumor tissue was extracted from each group (n = 03) and 100 mg of discarded tissue homogenized in 0.5 ml lysis buffer (0.1 m Tris-HCl pH = 7.6, and 0.1 m fresh dithiothreitol). After centrifugation at 10,000×*g* (1 min), equal amount of supernatant was added to the substrate-containing reaction buffer (0.1 m dithiothreitol and 5 µl of 4 mM DEVD-p-NA) and incubated for 120 min at 37 °C. Finally, the caspase-9 activity was assessed by the microplate reader (BioTek, 800TS, USA) at an absorbance of 405 nm. Each experiment was repeated in triplicate.

### Immunohistochemical analysis of TC-1 tumors

The transplanted TC-1 tumors were harvested, fixed in a 10% formaldehyde solution, embedded in paraffin, and cut into slices using standard procedures. After antigen retrieval (10 mM sodium citrate buffer, pH 6.0), endogenous peroxidases were blocked by 3% hydrogen peroxide in PBS for 10 min. Tissue sections were treated with primary antibodies for anti-cleaved caspase-3 (Abcam., Cambridge, UK) and mouse anti-HPV16 E6 (Abcam., Cambridge, UK) diluted at 1:500 concentrations in blocking buffer for 24 h at 4 °C.

Sections were then treated with horseradish peroxidase (HRP)-conjugated secondary antibodies at 1:100 dilutions and visualized using DAB plus chromogen substrate (Dako, Agilent, CA, USA) and hematoxylin counterstain. For quantification, five fields of view from at least four separate tissue sections were counted for each group (n = 03) using image J software. The number of positive brown-stained cells over the total number of cells was estimated and used to determine the percentage (%) of the staining area.

### Statistical analysis

All statistical analysis was performed using SPSS 16.0 software through one-way ANOVA technique. Probability values of *P < 0.05, **P < 0.01, and ***P < 0.001 were considered to demonstrate statistical significances.

## Results

### Lymphocyte proliferation

**Fig. 3:**
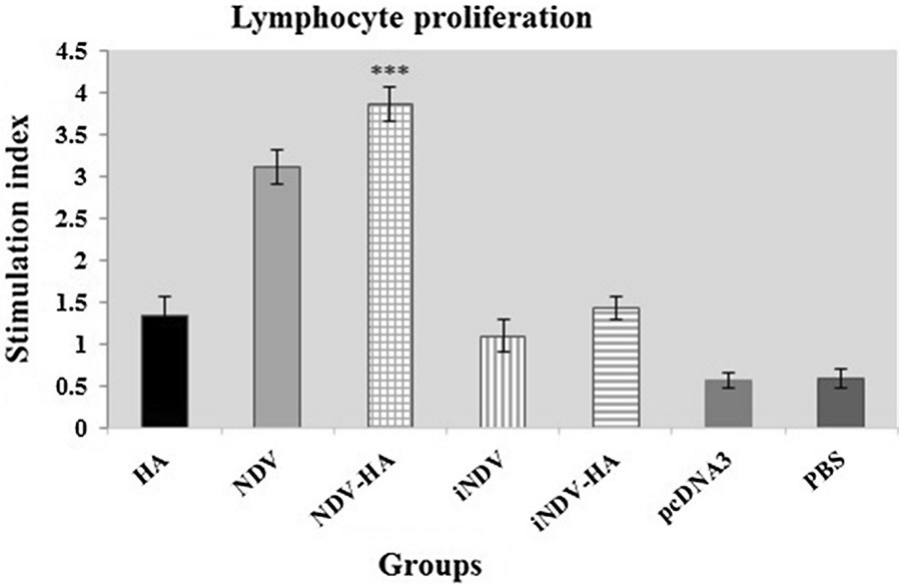
Lymphocyte proliferation assay. Lymphocyte proliferation was estimated and absorbance was measured at 540 nm. The stimulation index (SI) of splenic cells within different groups was calculated to determine the proliferation activity. The results indicate that NDV-HA2 and NDV groups stimulate significant differences when compared to iNDV-HA2, iNDV, HA, PBS and pcDNA3 control groups. In addition, the stimulation index was significant in iNDV-HA2, iNDV, and HA2 treated groups compared to pcDNA3 and PBS control groups. The results are representative of three independent experiments. ***P < 0.001

To determine whether the E7-specific lymphoproliferative response mainly resulted from NDV-HA2 treatment, Lymphocyte proliferation assay was performed among experimental groups. The mice treated with NDV-HA2 showed a significant lymphocyte proliferation response in comparison with the iNDV-HA2, HA2, iNDV, and iNDV groups and to a lesser extent in comparison with individual NDV (P < 0.001). Of note, a significant difference was observed between the NDV-HA2 and NDV groups in comparison to the pcDNA3 and PBS control groups (P < 0.001). Additionally, there was no noticeable expansion of splenocytes against E7 antigen from C57BL/6 mice treated with pcDNA3 and PBS control groups (Fig. 3). These results suggest that treatment with NDV-HA2 and NDV can significantly stimulate E7-specific T-cell responses.

### Cytotoxic T lymphocyte (CTL) assay

To gain more insight into the anti-tumor mechanism of the NDV-HA2, we designed an LDH release assay to indicate the cytotoxic activities of the CTLs induced by HPV-16 E7 epitope. Since the highest percentage of specific target lysis has been detected for E7-specific CTLs at an Effector: Target (EL4) ratio of 100:1, this ratio was selected for further analysis.

**Fig. 4:**
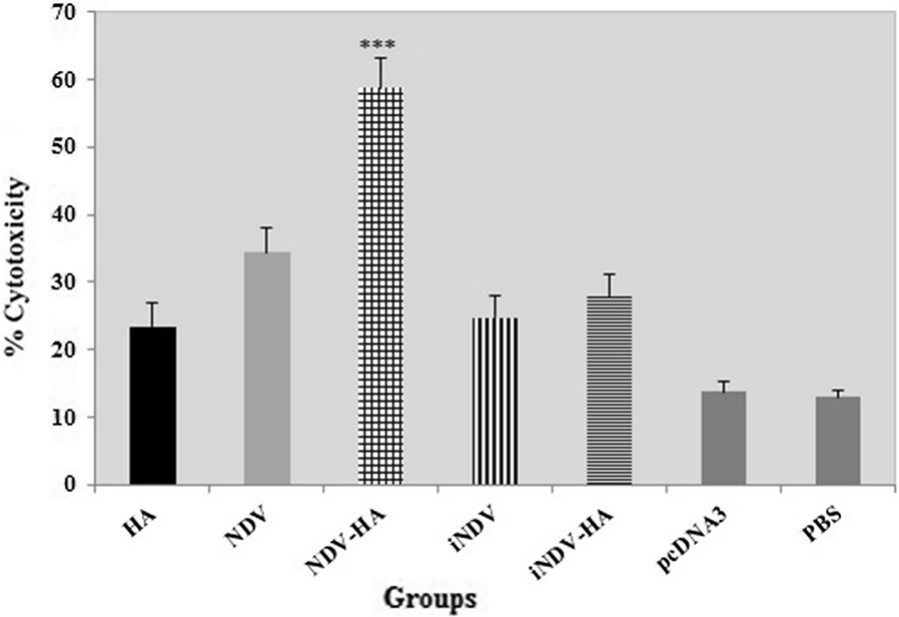
Cytotoxic T lymphocyte response following treatment with NDV-HA2. Results revealed that treatment with NDV-HA2 can significantly increase CTL responses compared with control groups (pcDNA3 and PBS groups). Also, cytolytic analysis of CTL lymphocytes demonstrated a greatly increased CD8^+^ activity in the group of mice treated with NDV-HA2 compared with the NDV group. Data points represent means ± SD of triplicate measurements. ***P < 0.001

The analysis of cytolytic activity showed that splenocytes of all treated groups significantly pulsed in response to specific antigen in comparison to pcDNA3 and PBS control groups (P < 0.001) (Fig. 4). Our results also illustrate that mice treated with NDV-HA2 showed significantly higher antigen-specific CTL responses compared to iNDV-HA2, HA2, iNDV, and iNDV groups (P < 0.001). Moreover, a significantly higher E7-specific lytic activity was detected in mice treated with NDV-HA2, as compared to mice treated with NDV (p < 0.001) (Fig. 4). As expected, no antigen-specific cytolytic response was observed for the C57BL/6 mice groups that had been treated with pcDNA3 and PBS (P > 0.001). Finally, the result revealed that the NDV-HA2 group could enhance the specific cytolytic responses against TC- 1 in the syngeneic model.

### Cytokine secretion and granzyme B expression assay

**Fig. 5:**
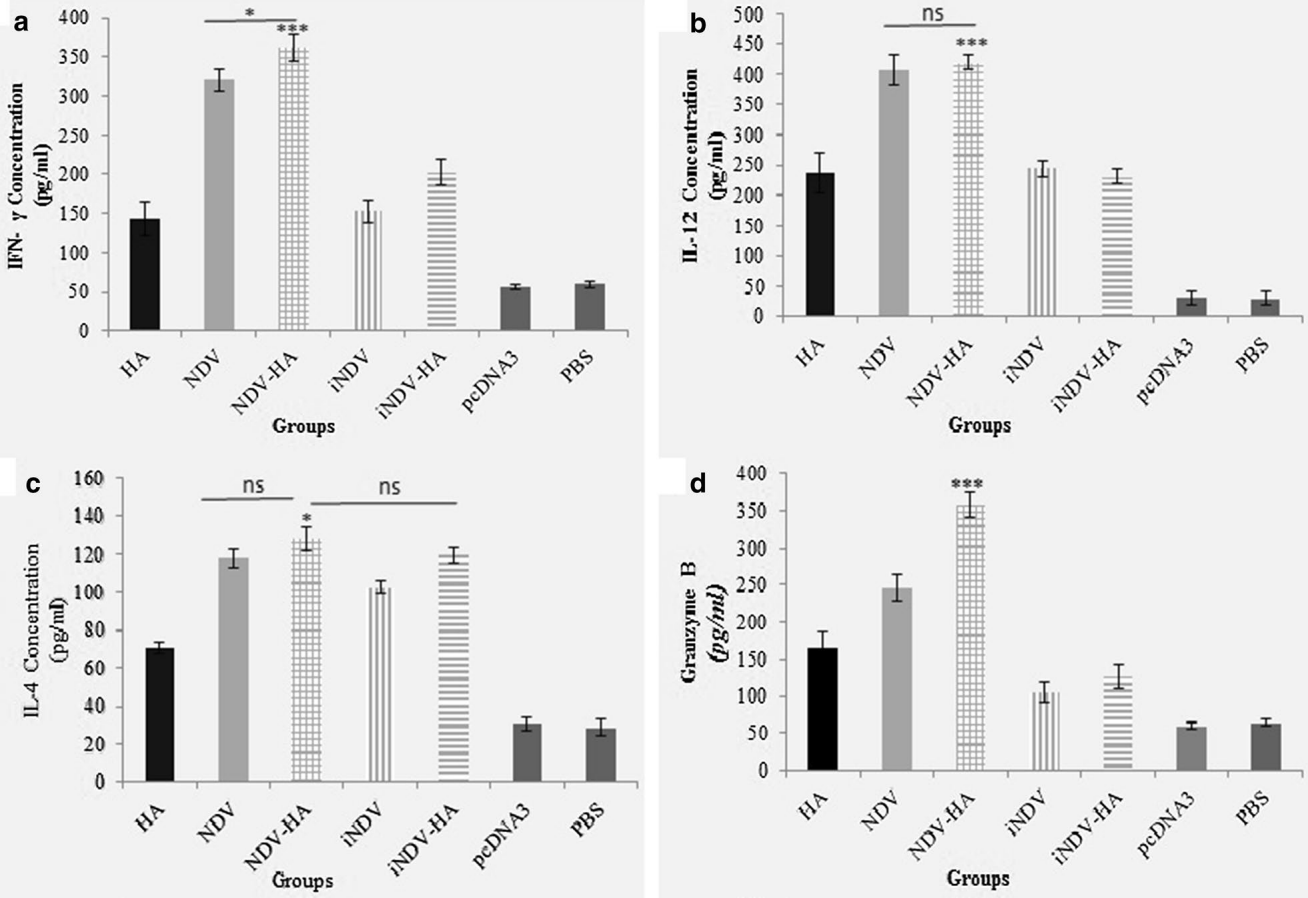
Cytokines (Interferon-γ (IFN-γ), interleukin (IL)-12, and IL-4) and granzyme B levels in spleen cell cultures of mice two weeks after second treatment determined by ELISA assay. **a** IFN-γ level was significantly higher (P < 0.001) among NDV-HA2 and NDV groups compared with all other groups. NDV-HA2 also showed a negligible increase for IFN-γ in comparison with NDV (P < 0.05). **b** IL-12 level in NDV-HA2 and NDV groups were significantly higher (P < 0.001) in comparison with other groups. **c** The distinction between IL-4 concentration of NDV-HA2, NDV, iNDV, and iNDV-HA2 was not significant and it was higher from HA2 and negative control groups (P < 0.001). **d** Granzyme B protein level was significantly more (P < 0.001) among NDV-HA2 treated mice compared to all other groups. The results are representative of three independent experiments. *P < 0.05, **P < 0.01, ***P < 0.001; ns, no significant difference

Cytokine assay was employed to monitor the balance of the adaptive immune response induced by NDV-HA2. As shown in Fig. 5a, b, splenocytes from both NDV-HA2- and NDV-treated mice induced a significant response of IFN-γ and IL-12 compared to iNDV-HA2, iNDV, and HA2 groups as well as control PBS and pcDNA3 ones (P < 0.001). Further investigation of expression profile showed that NDV-HA2 treatment significantly enhanced expression of IFN-γ in response to specific antigen stimulation in comparison to NDV-treated mice (P < 0.05), whereas IL-12 analysis showed no significant distinction between the two groups’ responses. These results suggest that NDV could induce Th1 cytokines that may play a critical role in strengthening the anti-tumor cellular immune system. Then, analysis of humoral cytokines showed that IL-4 expression was significantly increased in NDV-HA2, NDV, iNDV-HA2, and iNDV treated groups in comparison to HA2 alone and PBS or pcDNA3 control groups (P < 0.001) (Fig. 5c). Meanwhile, IL-4 analysis represented no significant response differences among NDV-HA2, NDV, and iNDV-HA2 groups, demonstrating the successful promotion of Th2 cell differentiation in all of these groups. In addition, the level of granzyme B, as the intracellular effector of target cell death and extracellular immune signals propagator, was significantly higher for the NDV-HA2-treated group when compared to other experimental or control groups (P < 0.001) (Fig. 5d). These data evidenced that humoral and cellular responses were highly modulated in NDV-HA2 treated groups.

### Intratumoral IL-10 and TGF-ß secretion Assay

**Fig. 6:**
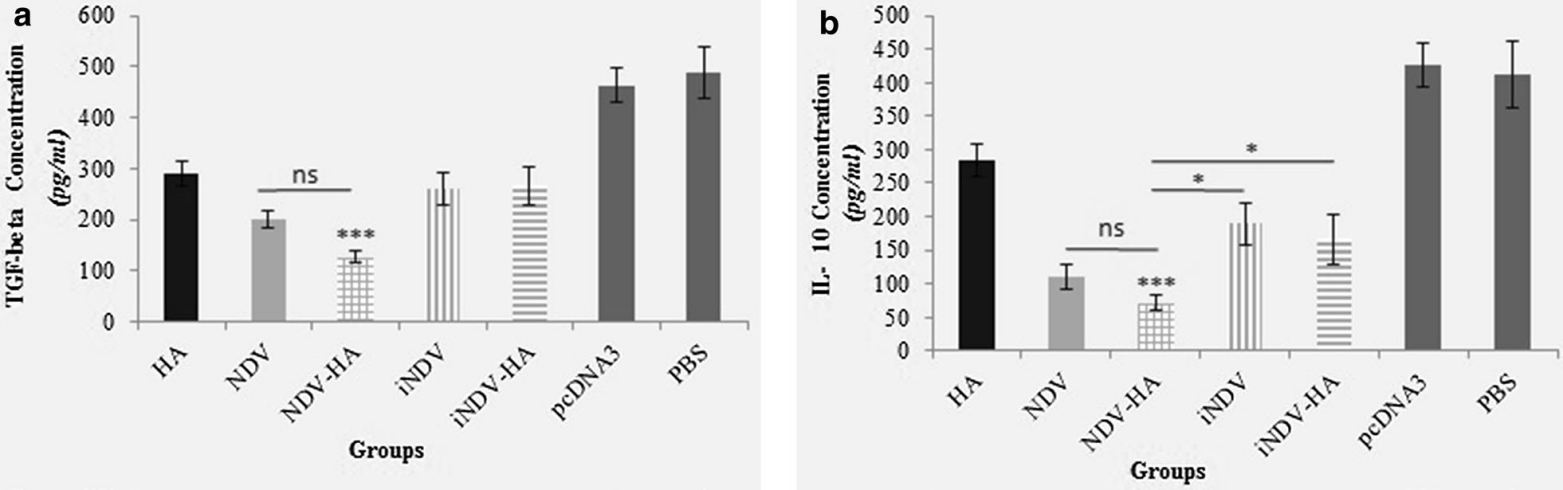
Cytokines (transforming growth factor-beta (TGF-β) and interleukin (IL)-10) levels in tumor lysate of mice two weeks after second treatment determined by ELISA assay. **a** The lowest level of TGF-ß was observed in NDV-HA2 and NDV groups (P < 0.001) compared to all other groups. **b** The level of IL-10 was significantly lower in NDV-HA2 and NDV (P < 0.001) in comparison with other groups. Data points represent means ± SD of triplicate measurements for three mice. *P < 0.05, ***P < 0.001; ns, no significant difference

In order to indicate whether our proposed therapy method could reduce the level of immunosuppressive cytokines, we measured IL-10 and TGF-ß responses in the tumor microenvironment. The results demonstrated that treatment with NDV-HA2 and NDV significantly suppressed the secretion of both IL-10 and TGF-ß in comparison with all experimental and control groups (P < 0.001) (Fig. 6). No significant difference was observed between NDV-HA2 and NDV groups in immunosuppressive cytokines expression. Other experimental groups including iNDV, iNDV-HA2, and to a lesser extent HA2 could suppress the secretion of IL-10 and TGF-ß compared to PBS and pcDNA3 control ones (P < 0.001). These observations prove the efficiency of NDV in the reduction of regulatory T cell (Treg) activity and subsequent induction of dendritic cells (DCs) and T cells function [23].

### Caspase-9 Activity

**Fig. 7:**
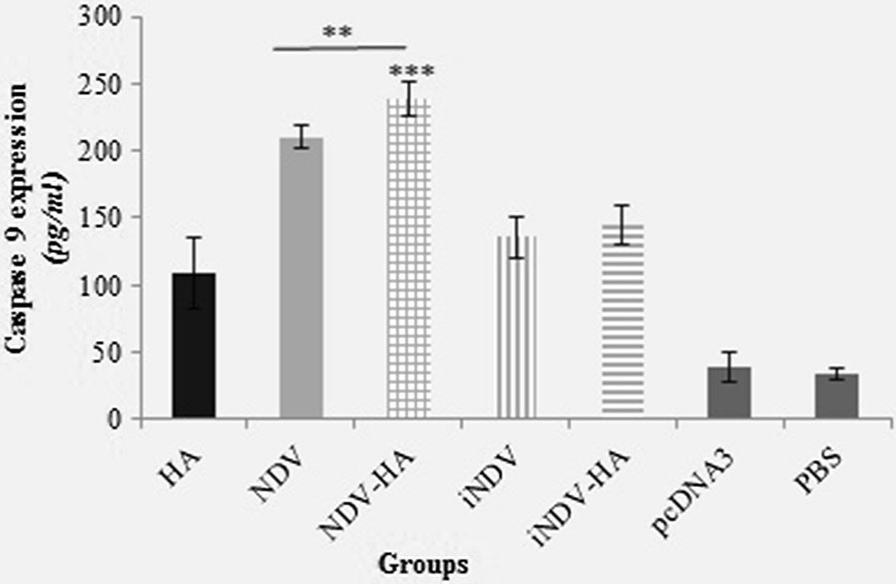
The expression level of caspase-9 protein in tumor lysate of treated groups was measured using the caspase ELISA kit. Treatment with NDV-HA2 significantly (P < 0.001) increased the level of Caspase-9 compared to control groups (PBS and pcDNA3) and also showed a higher level of caspase-9 (p < 0.01) compared with NDV group. Additionally, all other experimental groups induced a higher level expression of caspase-9 in comparison with control groups (p < 0.001). Data points represent means ± SD of triplicate measurements for three mice. **P < 0.01, ***P < 0.001

OVs directly induce a cytolytic effect on tumor cells or indirectly promote tumor cells apoptosis [24]. Our previous study has shown that oncolytic NDV induces apoptosis in the TC-1 cell line [3]. It has been proven that caspase 9 is the activator of other caspases and therefore mediates the apoptosis in several cancer types such as cervical cancer [25, 26]. In this regard, we investigated the effects of oncolytic NDV on the activation of intratumoral caspases-9 expression. We observed that oncolytic NDV highly induced the activation of caspase-9 in both NDV-HA2 and NDV groups compared to the other groups (P < 0.001) (Fig. 7). The results also illustrated that NDV-HA2 treatment significantly induced the expression of caspase-9 in tumor lysate in comparison to NDV-treated mice (P < 0.01). Taken together, our results indicate that the use of oncolytic NDV in combination with HA2 gene therapy could potentially induce apoptosis through the internal pathway.

### Immunohistochemical analysis

**Fig. 8:**
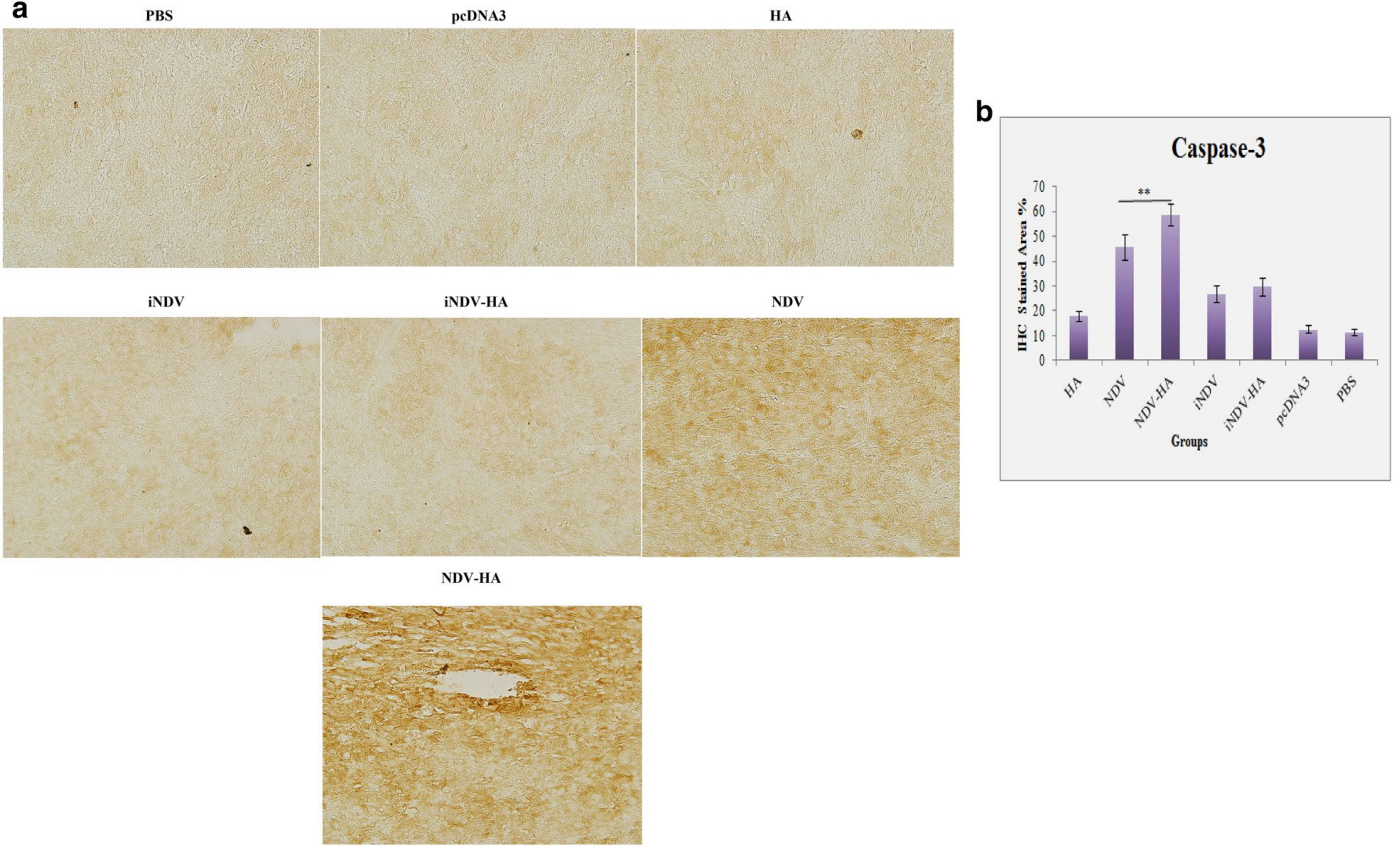
Immunohistochemistry of caspase-3 expression in tumor tissue. **a** Tumor-bearing mice were treated with NDV alone or in combination with HA2 plasmid then sacrificed and tumor tissue was excised to process for IHC studies. Randomly selected areas from each tumor were analyzed. Arrowheads represent immunohistochemical staining of caspase-3 (magnification: ×200). **b** Comparison between the stained areas of caspase-3 expression using Image J software. Bar graphs indicate the mean percentage (%) of staining area in tumor section of different groups. Values represent the mean ± standard deviation (SD) of three independent experiments. **P < 0.01

Wang et al. [27] have shown that caspase-3 plays a vital role in the effective function of virotherapy through suppression of IFN-α production and therefore enhancement of longevity and spread of oncolytic viruses in cervical cancer models. In another study, Hu et al. [28] have demonstrated that NDV apoptotic activity against lung cancer is initiated by an increase in caspase-3 activation. These investigations exemplify the potential of oncolytic viruses in the regulation of caspase-3 processing. Inspired by these results, and in order to explore the antitumor mechanism of NDV-HA2 on TC-1-induced HPV tumor, immunohistochemical analysis of cleaved caspase-3 expression in tumor tissues harvested on day 31 was assessed. The findings presented that a significant (P < 0.001) induction in caspase-3 expression was observed in tumors from mice treated with NDV and NDV-HA2 as compared to control groups. Furthermore, the analysis also showed that TC-1 tumor tissue sections from NDV-HA2 treatment have dramatically amplified staining of the apoptotic molecule caspase-3 expression as compared to NDV virotherapy (Fig. 8) (P < 0.01), indicating that combined NDV-HA2 therapy is able to stimulate key executioners of apoptosis as one of the central tumor growth inhibitory mechanism (Fig. 8).

**Fig. 9:**
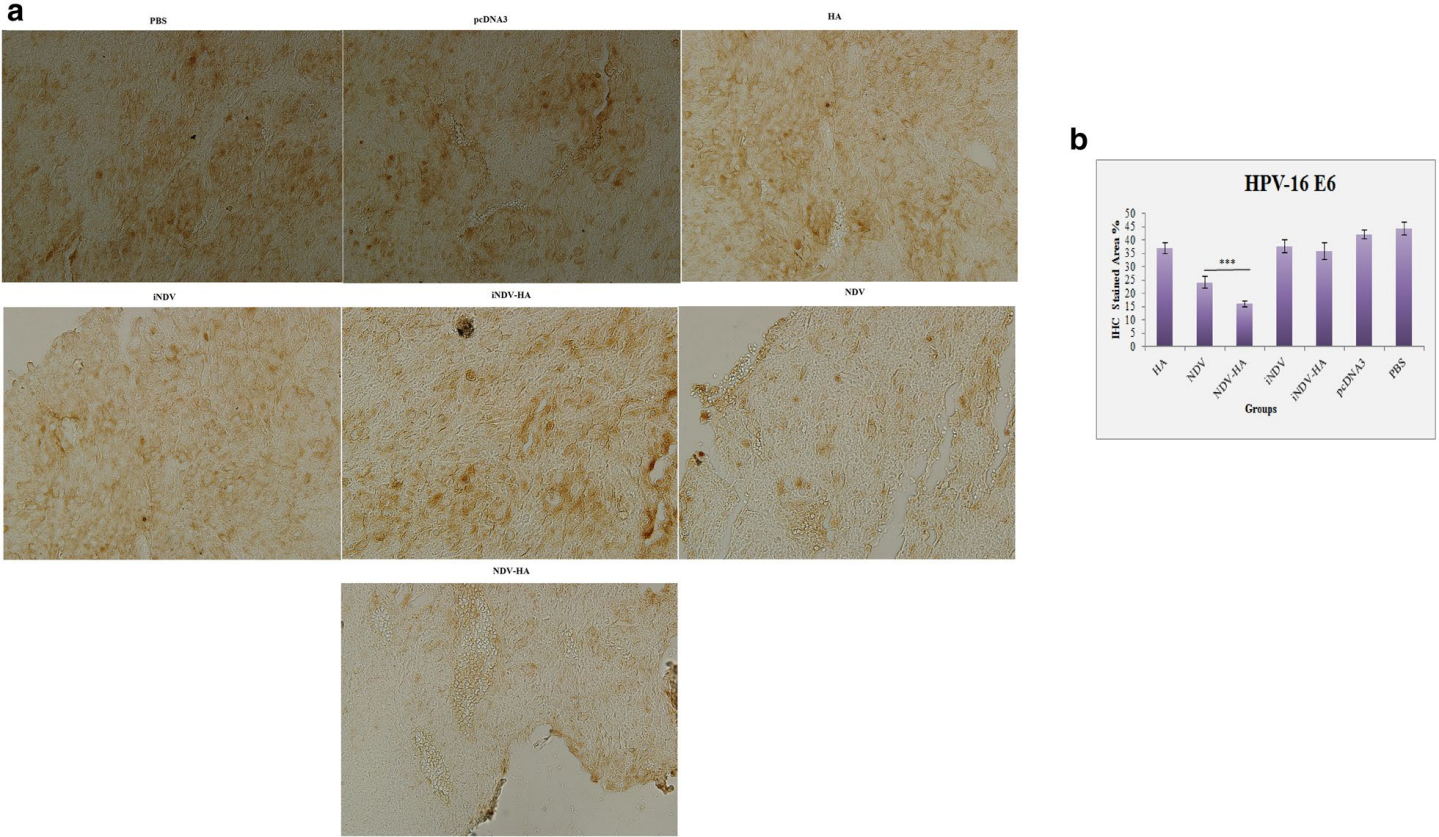
IHC analysis of HPV-16 E6 expression. **a** Treated mice were sacrificed and excised for IHC analysis. Randomly selected areas from each tumor were analyzed. **b** Comparison between the stained areas of E6 expression using Image J software. Bar graphs indicate the mean percentage (%) of staining area in tumor section of different groups. Values represent the mean ± standard deviation (SD) of three independent experiments. ***P < 0.001

HPV-16 E6 expression level, as onco-markers, was also evaluated as metrics of assessing antitumor properties in tumor tissues. The results presented in Fig. 9 demonstrate that, compared to the control cells, tumors from mice treated with NDV and NDV-HA2 down regulated the E6 expression (P < 0.001). Additionally, the analysis also presented that NDV-HA2 treatment significantly decreased E6 expression as compared to NDV virotherapy in TC-1 tumor tissue sections (P < 0.001). These findings indicate the action of combining HA2 gene therapy with oncolytic NDV virotherapy in decreasing tumor growth resulted from a reduction of E6 oncogene in the tumor tissue.

### Effect of PD-1 blockade on Ag-specific IFN-γ and IL-12 secretion

**Fig. 10:**
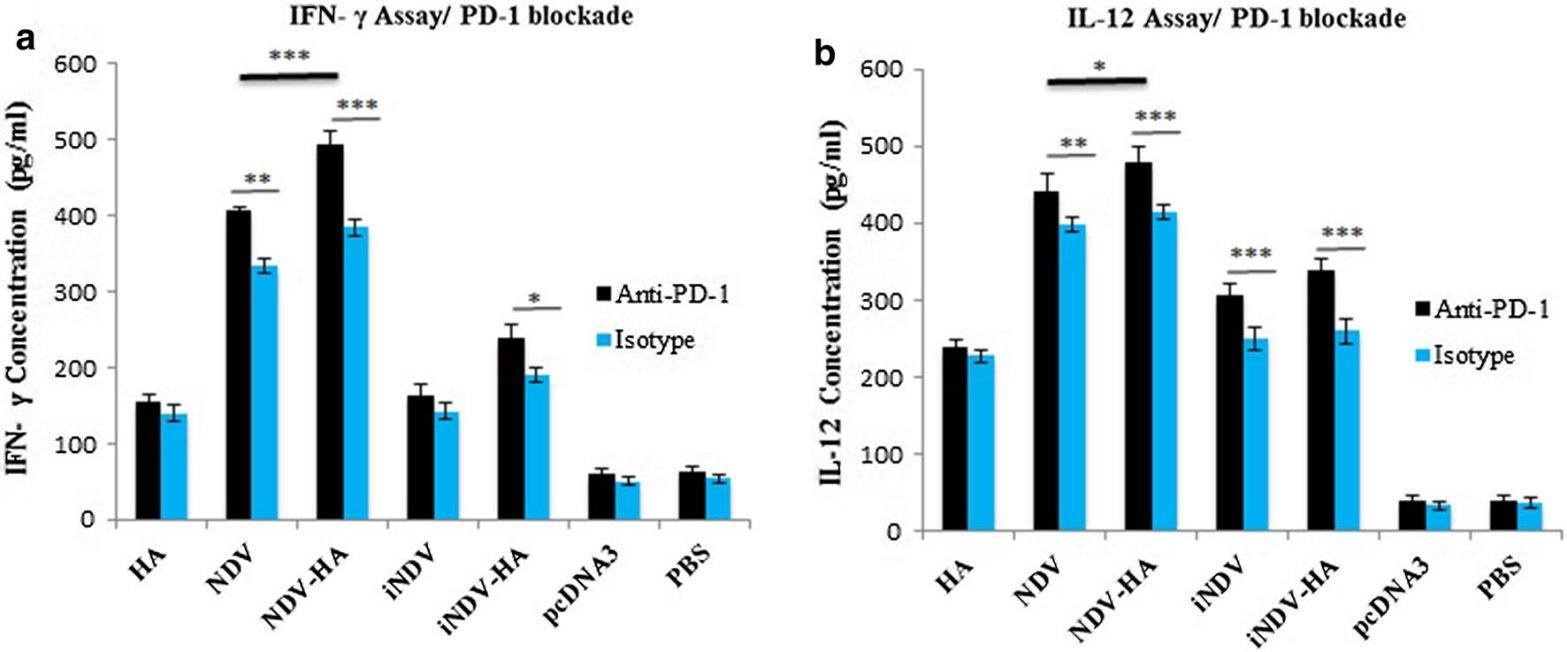
The impact of PD-1 blockade on the level of cytokine secretion using ELISA assay as described in “Methods” section. **a** IFN-γ level in NDV-HA2 and NDV groups was highly increased (P < 0.001) when coupled with PD-1 blockade. Except for iNDV-HA2, treatment with anti-PD-1 did not enhance the level of IFN-γ secretion among other groups. **b** The level of IL-12 secretion increased in NDV-HA2, iNDV-HA2, iNDV (P < 0.001), and NDV (P < 0.01) when treated with anti-PD-1 in comparison with isotype control groups. Data points represent means ± SD of triplicate measurements for three mice. *P < 0.05, **P < 0.01, ***P < 0.001

Recently, Garris et al. [29] could clarify the mechanism through which anti-PD-1 checkpoint blockade activates antitumor T cells *in vivo*. They showed that upon suppression of the PD-1 receptor, IFN-γ is upregulated and so stimulate DCs to secrete IL-12, which itself activates T-cell-mediated tumor cell destruction. Regarding these valuable findings and in order to determine whether blockade of PD-1 signaling during NDV-HA2 treatment could induce a greater IFN-γ and IL-12 responses, C57BL/6 mice were treated as before with concomitant administration of anti-PD-1 mAb or isotype control mAb one day prior to all of the treatments for a period of 2 weeks. Two weeks following the final blockade and treatments, we determined the E7-specific IFN- γ and IL-12 responses using ELISA assay (Fig. 10). Treatment with anti-PD-1 mAb significantly increased IFN-γ production in NDV-HA2 and to a lesser extent in NDV and iNDV-HA2 groups in comparison to those treated with control rat IgG2a (Fig. 10a). PD-1 blockade did not cause any significant increase in the level of IFN-γ among iNDV and HA2 groups in comparison with control groups. Moreover, IL-12 level showed a noticeable increase in NDV-HA2, NDV, iNDV-HA2, and iNDV groups when the PD-1 pathway was suppressed (Fig. 10b). While the level of IL-12 in isotype control groups of NDV-HA2 and NDV did not show any significant difference, IL-12 level in the NDV-HA2 group was greater than NDV one (P < 0.05) in presence of anti-PD-1.

### Anti-tumoral activity of NDV-HA2

Regarding the immunological responses (i.e., enhancement of CTL proliferation and regulation of cytokines production) and immunohistochemical effects (augmentation of apoptotic activity) of NDV-HA2 treatment, we expected that these findings could be correlated with the oncolytic potential of NDV-HA2 therapy in tumor growth suppression *in vivo*. Consequently, mice were examined and the growth of the tumor was measured by caliper for six weeks and the effect of NDV-HA2 on inhibition of tumor growth as well as reduction of tumor volume was calculated and compared with other experimental and control groups.

**Fig. 11:**
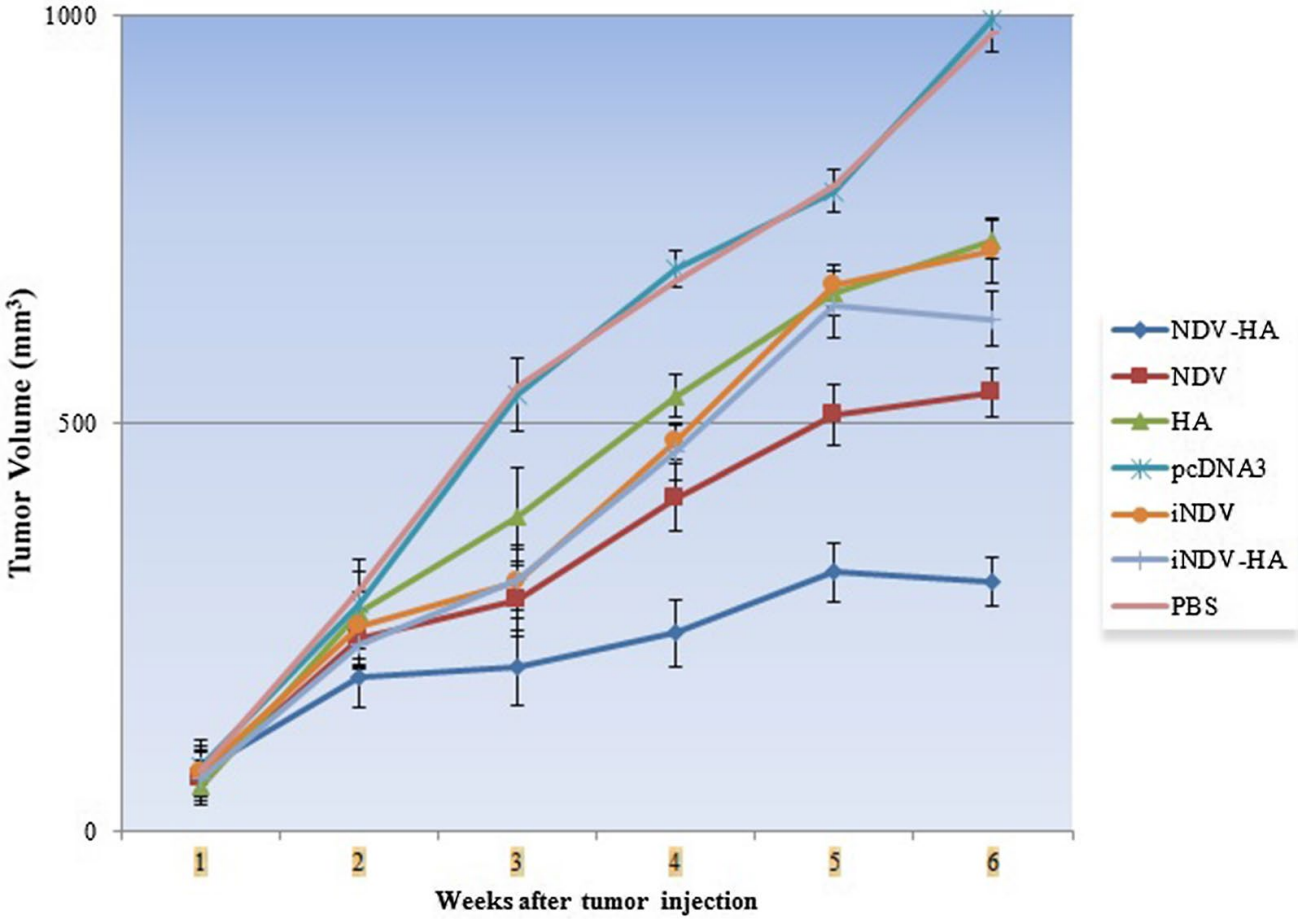
Growth curves of tumors in tumor-bearing mice treated with NDV-HA2, NDV, iNDV-HA2, iNDV, and HA2, or untreated control groups. Tumor volume was calculated using ocular checkup and palpation for seven mice per group three times a week for six weeks interval. Error bars represent mean ± SD for each group of mice. ***P < 0.001

As expected, the results proved that in all syngeneic mouse models treated with NDV-HA2, the tumor growth significantly abated compared to other groups (P < 0.001) (Fig. 11), which proves the complementary and effective role of HA2 gene therapy in anti-tumor activity of oncolytic NDV. Providing more explanation, the tumor size of the NDV-HA2 group in weeks 4, 5, and 6 was significantly lower compared with NDV alone (P < 0.001) and in all weeks compared to all other experimental and control groups (p < 0.001). The significant distinction between the activity of NDV-HA2 and NDV alone substantiates the critical role of HA2 in anti-tumor function of oncolytic NDV. Moreover, measurements revealed that in weeks 5 and 6, the tumor volumes of TC-1 bearing mice receiving NDV were significantly smaller than those treated with iNDV-HA2, iNDV, HA2, pcDNA3, and PBS groups (P < 0.001). Other experimental groups including iNDV-HA2, iNDV, and HA2 groups could also significantly control tumor growth compared to PBS and pcDNA3 control groups (P < 0.001), which approves the potentials of these therapeutic methods to somewhat induce apoptosis and antitumoral immune responses as shown in Fig. 11. Of note, HA2 also could improve the efficacy of inactivated NDV in tumor regression and tumor volume of the iNDV-HA2 group in week 6 was significantly lower than iNDV, HA2, and control groups (P < 0.001). Together, these data confirm the hypothesis that NDV has the potential to reduce tumor growth and its anti-tumor activity and therapeutic efficacy highly improves when combined with the HA2 gene as a supplement medication.

**Fig. 12:**
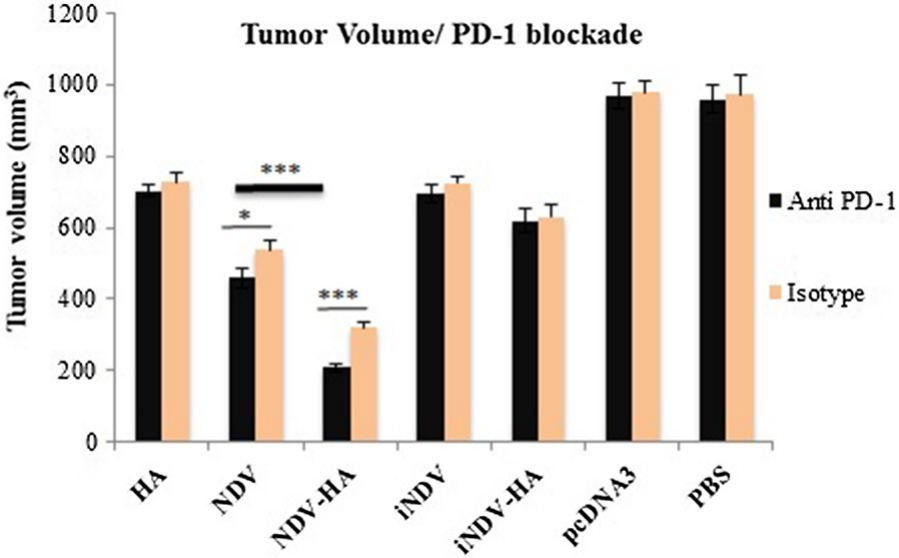
Comparison of ***in vivo*** anti-tumoral responses with or without PD-1 blockade. Tumor volume was calculated using ocular checkup and palpation for seven mice per group up to 6 weeks after tumor challenge among mice treated with anti-mouse PD-1 or isotype-matched IgG control. Error bars represent mean ± SD of triplicate measurements for seven mice. *P < 0.05, **P < 0.01, ***P < 0.001

In addition, in order to investigate the impact of PD-1 blockade on tumor regression, tumor volumes of C57BL/6 mice treated with rat anti-mouse PD-1 mAb (positive control) and rat IgG2a isotype (negative control) were measured six weeks after tumor challenge. The results showed that PD-1 blockade is only effective when is coupled with NDV or NDV-HA2 therapy and no significant tumor volume distinction was observed among other groups when compared with control isotype ones (Fig. 12). The findings indicated that the PD-1 pathway blockade could highly enhance the tumor regression in NDV-HA2-treating groups (P < 0.001) and to a lesser extent in TC-1 bearing mice treated with NDV (P < 0.05) compared to their equivalent control groups in the absence of anti-PD-1 antibody therapy. These observations assert the fact that the joint administration of HA2 gene therapy with NDV virotherapy and PD-1 blockade in the therapeutic mode significantly increased the protection of mice against TC-1- induced tumor challenge.

## Discussion

The urgent need for novel therapies of cancer has led to the emergence and development of a variety of biological approaches. In this regard, the employment of oncolytic viruses has made noticeable progress in the field of cancer therapy, both pre-clinical and clinical [30]. Due to their tumor selectivity and lack of evidence for developing resistance among cancer cells, oncolytic viruses have found a large therapeutic index and have shown promising results even as a cancer monotherapy option [31]. However, in order to guarantee and enhance the effectiveness of these approaches, they should be combined with other therapies [32]. Therefore, pre-clinical trials in order to find the best-matched therapy with a candidate oncolytic virus for each cancer type are needed. These trials have mostly focused on how the destruction of infected tumor cells or viral propagation among nearby malignant cells can be augmented. Since oncolytic viruses are able to infect only a portion of the cells within a tumor, the importance of bystander effect (through which neighboring uninfected cells are also killed) should be considered as a key element for designing novel combinatorial therapies [33]. One mechanism through which some viruses spread naturally is the expression of fusion proteins. Some of these viruses including NDV, Sendai virus (SV), respiratory syncytial virus (RSV), and measles are the best candidates for oncolytic virotherapy [13]. During infection, fusogenic viruses usually employ FMGs to constitute lethal multinucleated syncytia as a way for viruses to spread to nearby uninfected tumor cells [34]. Here, we revealed that the HA2 subunit of the influenza viral hemagglutinin protein as FMG fulfills this role perfectly. Regarding the bystander effect to improve the efficacy of oncolytic NDV virotherapy, we concentrated on the impacts of combining HA2 gene therapy with oncolytic NDV virotherapy in cervical cancer mice models. The results revealed that HA2 FMG is able to highly induce tumor regression and also promote the lymphocyte proliferation, CD8^+^ T cells cytotoxicity, IFN-γ and granzyme B induction, along with a decline in anti-inflammatory cytokines level. The immunohistochemical analysis also indicated that combined NDV-HA2 therapy is able to reduce key onco-markers, HPV-16 E6, as one of the central tumor growth inhibitory mechanism.

In agreement with our investigation and inspired by the evolutionary potential of fusogenic viruses’ FMGs, many studies have incorporated fusion proteins of distinct viruses with or without other therapeutic methods to increase the efficiency and safety of previously developed cancer therapies or develop a novel cancer therapy, respectively. Regarding the application of FMGs as an independent monotherapy technique, Galanis et al. [35] showed that Measles virus (MV) fusion (F) and hemagglutinin (H) proteins (MV-F and MV-H) and a recombinant form of the retroviral envelope protein of the gibbon ape leukemia virus (GALV.fus) are able to efficiently be employed in glioma gene therapy.

Harnessing the synergistic potential of FMGs, Hoffman et al. [36–39] have demonstrated that combining chemotherapy with FMGs of measles virus against colorectal and pancreatic cancer, respiratory syncytial virus against colorectal cancer, and vesicular stomatitis virus against colon cancer results in a noticeable enhancement in therapy results compared to individual chemotherapy. All these results can be attributed to the potential of viral FMGs to induce syncytia formation and subsequent activation of macrophages and DCs followed by IL-12-mediated priming of naive T cells and syncytial apoptosis or non-apoptotic autophagic-like cell death [16, 40, 41]. In another study, Linardakis et al. [42] showed that FMG-G of vesicular stomatitis virus is able to promote the anti-tumor efficacy of an allogenic melanoma vaccine.

When it comes to oncolytic virotherapy, many trials have tried to enhance the effectiveness of the method through manipulation of the viral fusogenic gene to obtain a hyperfusogenic-expressing virus or construction of recombinant virus expressing an adoptive FGM. For example, Nakamori et al. [17] revealed that construction of a recombinant oncolytic herpes simplex virus (HSV) by employment of both strategies, screening for a syncytial-optimized version of HSV and insertion of the hyperfusogenic FGM gene of gibbon ape leukemia virus among the viral genome, can be considered as a clinical method for treatment of advanced ovarian cancer.

In another strategy, FMGs are synergistically used with oncolytic viruses to augment their response against malign cells. In one study, Ahmed et al. [15] co-administrated the replicating adenovirus with a plasmid DNA encoding hyperfusogenic GALV FMG into the glioma and prostate cancer mice models. The results substantiated the high effectiveness of FMG in increasing the viral diffusion through the tumor mass, so that both reagents can efficiently be used at doses that they are ineffective individually.

NDV is among the most studied oncolytic viruses and stands out as an oncolytic virus that can be employed in most human cancers and is known for its good nature for human use [43]. These invaluable features let NDV be employed against a large number of cancers with promising results. It has been shown that NDV (LaSota strain) is able to induce antitumor cytotoxic effects by an increase in the production of TNF-α, IFN-γ, TRAIL, and granzyme B [18, 44]. Moreover, mechanistic studies of NDV’s way of apoptotic action have revealed the fact that NDV-mediated apoptosis is a caspase-dependent procedure in which the activation of caspase-9 through intrinsic pathway (mitochondrial related one) leads to the consecutive activation of caspase-3, which acts as the initiator of apoptosis and can be used as a well-suited biomarker to detect and quantify it [45, 46]. Recently, we [3] demonstrated that the LaSota strain of NDV vaccine could represent anti-tumor function against TC-1 cell line of HPV-associated carcinoma, expressing human papillomavirus 16 (HPV-16) E6/E7 oncoproteins. Our results proved the activation of early apoptotic pathways and increase in ROS secretion in infected tumor cells. However, the role of the bystander effect in spreading NDVs to neighboring uninfected cells and the formation of syncytia remained a question. Regarding the NDV-induced apoptosis, some studies have attributed this pathway to the cytotoxicity of viral syncytial formation [47]. Harnessing the fusion complex comprising both the viral fusion (F) and neuraminidase (HN) proteins, NDV is categorized among naturally occurring fusogenic oncolytic viruses, which can create syncytia between infected and neighboring uninfected cells [48]. It has been demonstrated that not only the fusion complex of NDV has a pivotal role in viral entry, but also it mediates the syncytia formation between infected and uninfected cells [49]. In order to enhance its oncolytic properties, engineered-NDV armed with hyperfusogenic FMG complex has been developed [50]. Surprisingly, this modified oncolytic virus didn’t show any increase in toxicity to healthy hepatic parenchyma cells, proving the fact that while its tumor cell destruction has been accelerated, NDV is able to preserve its specific tropism for tumor cells. Alternatively, it can be concluded that the incorporation of other viral fusogenic membrane proteins may result in the improvement of NDV virotherapy effectiveness.

For the first time, we employed influenza HA2 FMG as a complementary reagent beside oncolytic virotherapy. The only report for the deployment of HA-2 as an adjuvant for cancer therapy backs to the study by Michiue et al. [14]. Their study demonstrated that HA2 facilitates the penetration of p53 protein into the nucleus of glioma cells, improving its function in the provocation of P21WAF1 transcriptional activity. However, our unprecedented investigation revealed the ability of HA2 FMG in the promotion of anti-tumor responses of oncolytic viruses, particularly NDV virotherapy against HPV-associated cervical cancer.

Moreover, we explored to what extent PD-1 immune checkpoint blockade can enhance the anti-tumor efficacy of our suggested therapy. In this context, the results showed more than 30% decrease in tumor volume and a noticeable increase in the secretion of IL-12 and IFN-γ cytokines after the adjunction of anti-PD1. Previously, we [6] substantiated that PD-1 blockade is able to possess strong synergistic effects in combination with DNA vaccine encoding HPV-16-E7 antigen for the treatment of HPV-associated cancers. Very recently, Zamarin et al. [51] showed that NDV-injection upregulates the PD-L1 in the tumor microenvironment, hampering the oncolytic virus from attaining to its maximum efficiency in complete rejection of tumor cells. This report discloses the importance of incorporation of anti-PD-1 or (and) anti-PD-L1 blockade as an adjuvant for oncolytic NDV therapy.

## Conclusions

In this study, we indicated that the HA-2 subunit of influenza HA FMG can synergistically enhance the oncolytic NDV virotherapy efficacy against HPV-associated cervical cancer. This combinatorial therapy resulted in noticeable tumor-regression and down regulation of HPV-16 E6 onco-marker in response to an increase in cytotoxic CD8 T lymphocytes. Furthermore, the adjunction of the anti-PD-1 blockade to our proposed therapy led to the augmentation of the anti-tumor effect of NDV-HA therapy. These results clearly prove that the employment of FMGs as an assisting reagent along with omitting the PD-1 inhibitory pathway can highly improve the oncolytic properties of NDV in the treatment of cancers.

## Data Availability

Not applicable.

## Abbreviations

HA2: Hemagglutinin 2
NDV: Newcastle disease virus
FMGs: Fusogenic membrane glycoproteins
PD-1: Programmed death-1
EID50: Embryo Infectious Dose 50
FBS: Fetal bovine serum
IHC: Immunohistochemical
LPA: Lymphocyte proliferation assay
CTL: Cytotoxic T lymphocyte
LDH: Lactate dehydrogenase
OD: Optical density
IL-10: Interleukin-10
TGF-β: Transforming growth factor-β
HRP: Horseradish peroxidase
Treg: Regulatory T cell
DCs: Dendritic cells
SV: Sendai virus
RSV: Respiratory syncytial virus
MV: Measles virus
F: Fusion
H: Hemagglutinin
HSV: Herpes simplex virus
HPV-16: Human papillomavirus 16
