# Correction to: Synergy between hemagglutinin 2 (HA2) subunit of influenza fusogenic membrane glycoprotein and oncolytic Newcastle disease virus suppressed tumor growth and further enhanced by Immune checkpoint PD-1 blockade

**DOI:** 10.1186/s12935-020-01610-3

**Published:** 2020-10-28

**Authors:** Seyed Mohammad Miri, Mir Saeed Ebrahimzadeh, Elahe Abdolalipour, Mahsa Yazdi, Hassan Hosseini Ravandi, Amir Ghaemi

**Affiliations:** 1 Department of Virology, Pasteur Institute of Iran, Tehran, Iran; 2 Department of Microbiology, Golestan University of Medical Sciences, Gorgan, Iran; 3 Shefa Neuroscience Research Center, Khatam Alanbia Hospital, Tehran, Iran

## Correction to: Cancer Cell Int (2020) 20:380 10.1186/s12935-020-01476-5

Following publication of the original article [1], we were notified the Department of Virology, Pasteur Institute of Iran should have been mentioned as the only affiliation for the first, third and last author. The revised affiliation is reflected in this correction article.
